# Radiation Necrosis – A Growing Problem in a Case of Brain Metastases Following Whole Brain Radiotherapy and Stereotactic Radiosurgery

**DOI:** 10.7759/cureus.2037

**Published:** 2018-01-08

**Authors:** Yee Pei Song, Rovel J Colaco

**Affiliations:** 1 Clinical Oncology, ‎The Christie NHS Foundation Trust; 2 Radiation Oncology, ‎The Christie NHS Foundation Trust

**Keywords:** stereotactic radiosurgery, radiation necrosis, alk-positive adenocarcinoma, brain metastasis, non-small cell lung cancer, srs, laser interstitial thermotherapy, immunotherapy

## Abstract

Stereotactic radiosurgery (SRS) provides excellent control in the treatment of brain metastases (BM). The use of newer, targeted and immunotherapy treatments have resulted in improved overall survival in patients even with an extensive metastatic disease. Hence, it is increasingly important to consider the potential for late toxicities like radiation-induced necrosis (RN) of the brain. We present a case of a patient with stage IV anaplastic lymphoma kinase (ALK) positive adenocarcinoma of the lung who underwent stereotactic radiosurgery to her brain metastases and received targeted treatment. While her intracranial and extracranial disease remained well controlled, we discuss the radiation-induced necrosis she suffered as a result of the treatment, the related diagnostic dilemma involved, and the subsequent management of this late toxicity of stereotactic radiosurgery.

## Introduction

Stereotactic radiosurgery (SRS) is an established and effective treatment for brain metastases (BM), with excellent control rates, especially in the treatment of lesions with a maximum dimension of 2 cm or smaller [1]. Unfortunately, despite the improvement in disease control and overall survival it accords, it is not without toxicities. We present a case of a patient with multiple BM surviving 30 months with controlled BM following treatment with SRS, but whose main issues revolve around the radiation-induced necrosis (RN) she sustained as a result of the life-prolonging SRS.

## Case presentation

**Figure 1:**
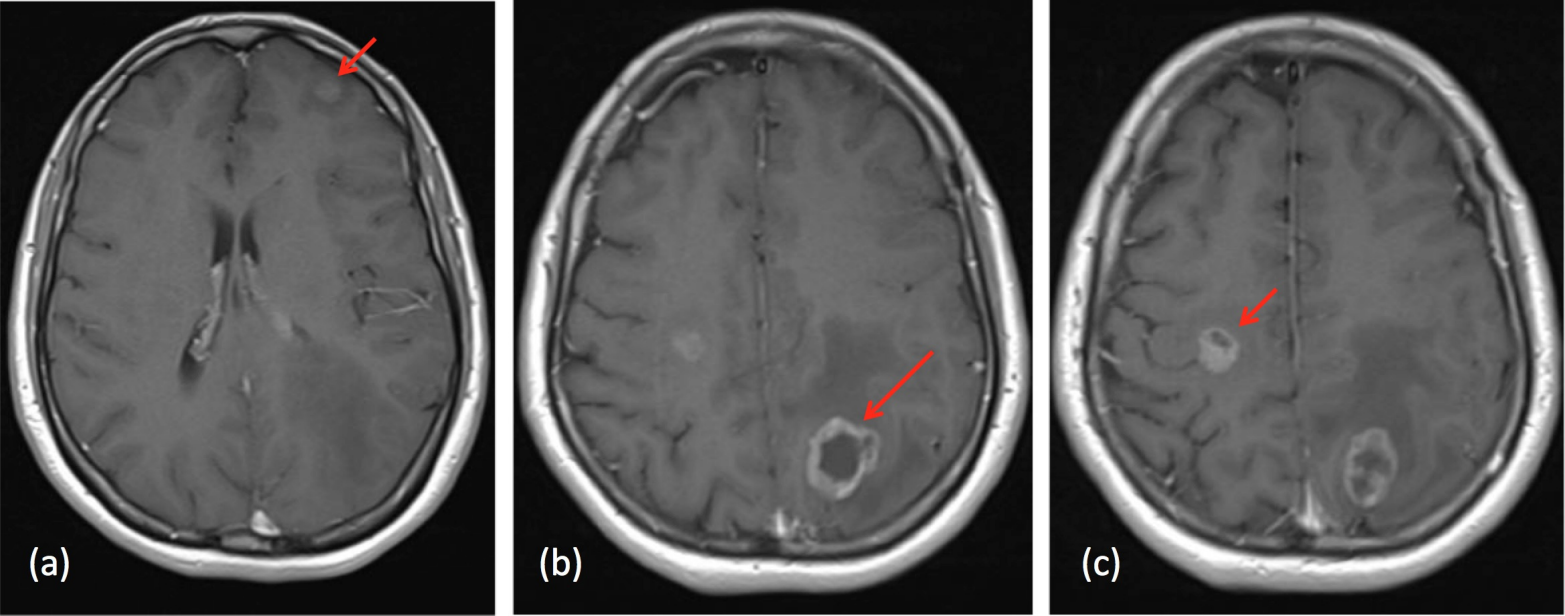
MRI head at diagnosis (November 2014) (a) 11 x 9.9 x 9.9 mm left frontal BM; (b) 24.3 x 25.3 x 22.2 mm left parietal BM; (c) 14.3 x 11.7 x 17.3 mm right frontal BM MRI: magnetic resonance imaging; BM: brain metastases

A 37-year-old female presented to the clinic in November 2014 with an unremitting upper respiratory tract infection. Further investigations diagnosed a T3N2 anaplastic lymphoma kinase (ALK) positive adenocarcinoma of the lung. She was found to have three brain metastases (Figure 1) and was treated by a local radiation oncologist with whole brain radiotherapy (WBRT) at a dose of 20 Gy in five fractions in November 2014.

**Figure 2:**
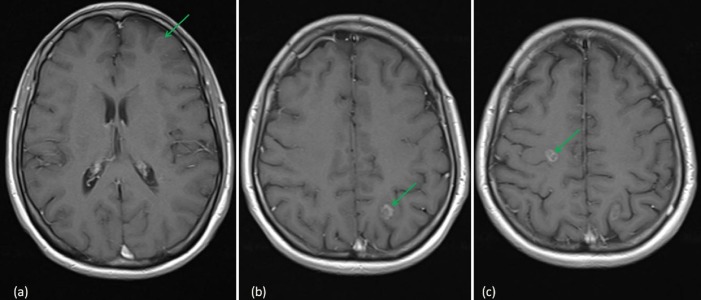
MRI head following WBRT (March 2015) (a) 7.3 x 6.8 x 5.2 mm left frontal BM; (b) 9.6 x 9 x 10.7 mm left parietal BM; (c) 9.4 x 7 x 11.5 mm right frontal BM MRI: magnetic resonance imaging; BM: brain metastases; WBRT: whole brain radiotherapy

She subsequently commenced systemic treatment with the ALK inhibitor, ceritinib, on a clinical trial. Follow-up magnetic resonance imaging (MRI) scans of the head in January and March 2015 showed an improvement in the brain metastases with a reduction in tumor size (Figure 2).

Further imaging studies in April 2015 demonstrated an increase in the size of her previously treated brain metastases with no new lesions. Her primary lung lesion continued to respond to treatment. She was asymptomatic from her brain lesions and was in good general condition with a Karnofsky Performance Status (KPS) of 90%. She underwent stereotactic radiotherapy to the three lesions in early May 2015. Each lesion was treated with 21 Gy in a single fraction to the 80% isodose using linear accelerator-based SRS. The 21 Gy isodose volumes and Radiation Therapy Oncology Group (RTOG) conformity indices (CI) were as follows - left frontal BM 1.69 cm^3^ (CI 1.69), left parietal BM 4.36 cm^3 ^(CI 1.55), and right frontal BM 4.35 cm^3 ^(CI 1.45). Follow-up MRI scans showed a stable disease.

**Figure 3:**
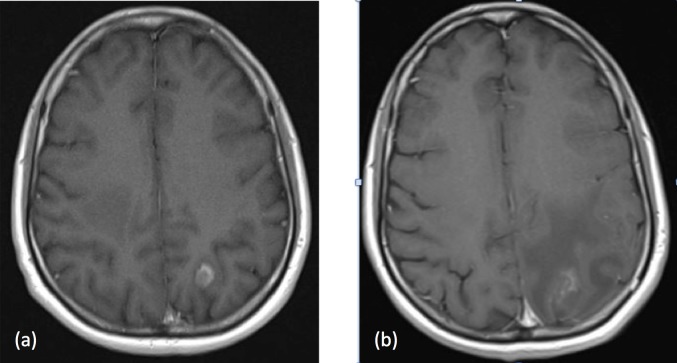
MRI head with a change in the left parietal lesion (a) Prior to SRS (May 2015): 10.2 mm x 12.4 mm; (b) Eight months following SRS (January 2016): 24.9 mm x 16.2 mm with peri-lesional edema MRI: magnetic resonance imaging; SRS: stereotactic radiosurgery

Eight months following SRS, she had a single episode of grand mal seizure, and imaging at this time showed an increase in the size of a previously treated left parietal lesion (Figure 3). Her scans and history were reviewed in three separate independent tumor boards and all were in agreement with a diagnosis of radiation-induced necrosis. 

**Figure 4:**
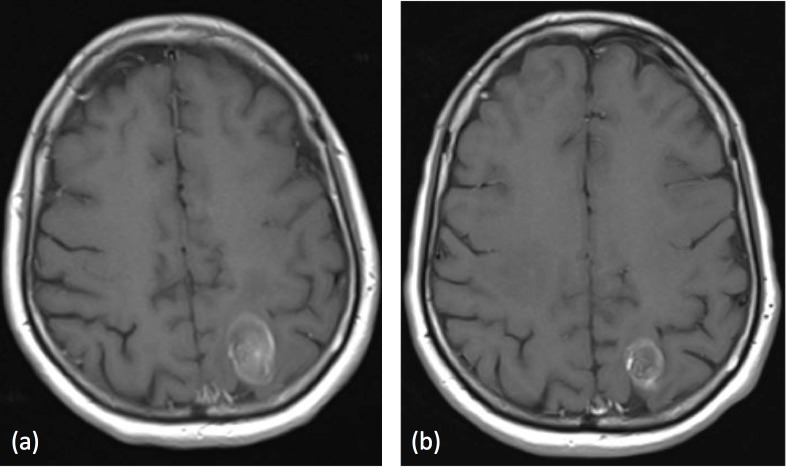
MRI head following LITT (a) Left parietal lesion measuring 28.3 x 18.5 x 20.3 mm six weeks post-LITT; (b) Left parietal lesion measuring 19.4 x 15.1 x 20.1 mm six months post-LITT MRI: magnetic resonance imaging; LITT: interstitial thermal therapy

The patient was initially treated with anti-epileptics and high-dose steroids with no significant shrinkage of the enlarging lesion. She subsequently underwent laser interstitial thermal therapy (LITT) to the left parietal lesion in late January 2016. Follow-up MRI scans showed an initial increase in peri-lesional edema, but this resolved and the lesion decreased in size (Figure 4).

In July 2016 (15 months post-SRS), further imaging found an increase in size and a change in the morphology of the previously treated right frontal lobe lesion, which could signify disease progression or further radiation-induced necrosis. There was also the development of a new 5 mm lesion in the left frontal lobe. Following a course of steroids, the previously enlarging right frontal lesion reduced in size with no further progression of other intracranial lesions. Her extracranial disease remained stable, and it was decided to switch her ALK-inhibitor to brigatinib.

Unfortunately, she developed neurological symptoms with a weakness of her left upper limb in October 2016. Once again, the diagnosis was felt to be radiation-induced necrosis and an MRI scan confirmed a further increase in the size of the right frontal lobe lesion while all other lesions, including the previously laser ablated left parietal lesion, remained relatively stable. She was once again commenced on steroids and reported improved left upper limb power shortly after.

A follow-up MRI scan of the head showed a continued increase in the size of the right frontal lesion while other intracranial lesions remained stable, and CT scans showed that her extracranial disease continued to improve. Steroid treatment was restarted and while there was a good symptomatic response, the symptoms recurred when the steroid dose was reduced or stopped.

The response to steroids and the incongruity of other known lesions remaining stable or reducing in size supported the diagnosis of radiation-induced necrosis. She underwent further LITT treatment to the right frontal lesion in March 2017. As with her initial post-treatment scan in 2016, her MRI scan shortly after treatment showed an increase in peri-lesional edema. Further MRI scans of the head showed no further worsening of the intracranial disease, and she remained clinically asymptomatic. At last scan, the patient remained clinically and radiologically stable 30 months post BM diagnosis with her main symptoms being minor right arm weakness and a dragging of her right leg related to her radiation-induced necrosis and the subsequent treatment of this. Her systemic disease remains controlled on brigatinib.

## Discussion

Approximately 25% of patients with advanced non-small cell lung cancer (NSCLC) may develop BM and the incidence continues to rise [2]. Stereotactic radiosurgery (SRS) is an increasingly popular treatment for brain metastases, with excellent control rates reported for lesions treated [3]. With increased radiological surveillance of patients with BM coupled with longer survival in patients treated with SRS in the modern era, the identification of radiological changes in BM following treatment with SRS is an increasing challenge. In particular, the differentiation between radiation-induced necrosis (RN) versus tumor progression is a growing problem [4]. Positron emission tomography (PET) and MRI spectroscopy have been used in the evaluation of enlarging lesions following SRS. However, these imaging modalities are neither sensitive nor specific for the diagnosis of RN and are not routinely used. 

RN is an inflammatory reaction that occurs between six and 24 months following SRS. RN appears as contrast-enhancing lesions with peri-lesional edema at the site of previous SRS radiologically and can be asymptomatic or cause neurological symptoms. Commonly cited risk factors for RN include target dose and volume, previous radiotherapy, and the concurrent use of systemic agents. In the case presented, the patient underwent whole brain radiotherapy six months prior to SRS, and the use of targeted therapy agents (ceritinib and brigatinib) may also have contributed to a greater risk of RN. In a study of 180 patients, Colaco et al. observed higher rates of RN in patients who received both SRS and immunotherapy or targeted therapies compared to those who had SRS alone or with concurrent cytotoxic chemotherapy [5]. Another study comparing the rates of RN in melanoma patients who underwent SRS alone with those who were treated with SRS and BRAF inhibitors have also shown an increase in the occurrence of RN in those who received BRAF inhibitors [6].

Immunotherapy and targeted therapy has changed the landscape of cancer and improved the prognostic outlook of patients with ALK-positive NSCLC. A recent multi-institutional publication reported a median overall survival of 49.5 months after the development of brain metastases in patients with ALK-positive NSCLC [7]. With the increasing use of immunotherapy and targeted therapy for a variety of cancer diagnoses, including malignant melanoma and breast and lung cancers, the potential synergistic effect of these treatments with SRS increases the dilemma that radiation oncologists face in evaluating growing lesions post-SRS.

In this case, the patient was diagnosed with stage IV NSCLC almost three years previously. She remained largely asymptomatic of her systemic disease and successive imaging studies continue to show a radiologically stable disease extracranially. While it is likely that she would eventually develop neurological symptoms if the BM was left untreated, she had no neurological symptoms prior to either WBRT or SRS. Her subsequent neurological symptoms were due to the effect of RN as opposed to a progressive intracranial disease as evidenced by multiple brain MRI scans and response to RN treatments. The toxicities of cancer treatment are often more problematic than the disease itself and, in this case, the cost of survivorship is right limb weakness and the resulting functional problems.

The diagnosis of RN is challenging and differentiating this from intracranial disease progression often poses a diagnostic dilemma. As we have shown in this case, the symptomatic and radiological presentations are very similar. A misdiagnosis of disease progression could lead to referral for open surgery or further SRS, which would likely worsen RN. It could also impact the patient’s cancer treatment in general and result in the early termination of a systemic treatment, which the patient may be responding to. It is vital that clinicians are aware of the risk factors of developing RN and consider this as an important differential diagnosis in relevant patients.

The management of RN also poses many challenges. Options for treatment range from conservative management in asymptomatic patients, to medical management, hyperbaric oxygen, and surgical intervention. The mainstay of treatment in a symptomatic patient is corticosteroids at the lowest possible dose until the lesion and symptoms resolve. As in the case of the patient presented, this often translates to a long course of steroids, which often brings medication-related side effects. There is a suggestion that the upregulation of vascular endothelial growth factors (VEGF) may be involved in RN. This makes VEGF-inhibitor therapy a potential treatment option. There is now evidence supporting the use of bevacizumab, a humanized monoclonal antibody, in the treatment of symptomatic RN that did not respond adequately to corticosteroids [8].

In patients who have an inadequate response to medical treatment, surgical removal will then need to be considered. Apart from the risk of neurocognitive and functional loss with open surgery, there is also the disruption of systemic anti-cancer treatment during the long recovery period. Minimally invasive techniques, such as laser interstitial thermal therapy (LITT) pose a suitable alternative and should be considered. This magnetic resonance (MR)-guided procedure allows a neurosurgeon to use a stereotactically guided laser to generate hyperthermia across the lesion through a small incision, resulting in controlled cell death [9]. Compared to open surgery, patients who undergo this procedure have a shorter inpatient stay and a faster recovery.

Hyperbaric oxygen (HBO) therapy improves angiogenesis in hypoxic tissues and is an option for patients who have contraindications to medical and surgical interventions. The side effects are mild and reversible. A symptomatic response has been observed in almost 60% of patients, with a similar reduction in steroids requirements [10].

## Conclusions

We present a case of excellent disease response and long-term survival (over 30 months) following SRS for BM in a patient whose only ongoing symptoms relate to RN rather than intracranial or extracranial disease progression. This case highlights the importance of considering potential risk factors for RN, including the type of systemic therapy and the significant survivorship impact that RN may have on patients treated with SRS for BM. Furthermore, as anti-cancer treatments evolve and patient survival improves, the evaluation of the growing lesion post SRS is likely to become an increasingly important challenge in the future. It is crucial for RN to be considered as a differential diagnosis and patients managed appropriately.
